# Circovirus in Tissues of Dogs with Vasculitis and Hemorrhage

**DOI:** 10.3201/eid1904.121390

**Published:** 2013-04

**Authors:** Linlin Li, Sabrina McGraw, Kevin Zhu, Christian M. Leutenegger, Stanley L. Marks, Steven Kubiski, Patricia Gaffney, Florante N. Dela Cruz Jr, Chunlin Wang, Eric Delwart, Patricia A. Pesavento

**Affiliations:** Blood Systems Research Institute, San Francisco, California, USA (L. Li, E. Delwart);; University of California, San Francisco (L. Li, E. Delwart);; University of California School of Veterinary Medicine, Davis, California, USA (S. McGraw, K. Zhu, S.L. Marks, S. Kubiski, P. Gaffney, F.N. Dela Cruz Jr, P.A. Pesavento);; IDEXX Laboratories, West Sacramento, California, USA (C.M. Leutenegger);; Stanford Genome Technology Center, Stanford, California, USA (C. Wang)

**Keywords:** circovirus, vasculitis, deep sequencing, viruses, dogs, hemorrhagic gastroenteritis, necrotizing vasculitis, granulomatous lymphadenitis, canine vascular disease

## Abstract

We characterized the complete genome of a novel dog circovirus (DogCV) from the liver of a dog with severe hemorrhagic gastroenteritis, vasculitis, and granulomatous lymphadenitis. DogCV was detected by PCR in fecal samples from 19/168 (11.3%) dogs with diarrhea and 14/204 (6.9%) healthy dogs and in blood from 19/409 (3.3%) of dogs with thrombocytopenia and neutropenia, fever of unknown origin, or past tick bite. Co-infection with other canine pathogens was detected for 13/19 (68%) DogCV-positive dogs with diarrhea. DogCV capsid proteins from different dogs varied by up to 8%. In situ hybridization and transmission electron microscopy detected DogCV in the lymph nodes and spleens of 4 dogs with vascular compromise and histiocytic inflammation. The detection of a circovirus in tissues of dogs expands the known tropism of these viruses to a second mammalian host. Our results indicate that circovirus, alone or in co-infection with other pathogens, might contribute to illness and death in dogs.

Circoviruses are nonenveloped, spherical viruses with a single-stranded circular DNA genome of ≈2 kb; they group as a genus within the family *Circoviridae*, together with the proposed genus *Cyclovirus* and the phylogenetically more distinct genus *Gyrovirus* (*1*). Most of the known species in the genus *Circovirus* infect birds and cause signs including malformations and necrosis of the integument, lymphoid depletion, and immunosuppression (*2*).

Before 2012, the only circoviruses reported to infect mammals were the 2 closely related porcine circoviruses (PCVs) (*3*). PCV2 is the primary pathogen associated with a spectrum of swine diseases called porcine circovirus–associated diseases that have been described in pigs worldwide. PCV2 infection causes severe economic losses because of increased mortality and reduced production, making it one of the most economically important viruses in the global swine industry. Among lesions that have been attributed to PCV2 infection are pneumonia, enteritis, lymphadenitis, vasculitis, nephritis, and reproductive disease (*4*). In cases for which PCV2 is considered causative, immunohistochemical and in situ hybridization (ISH) analyses demonstrate large amounts of PCV2 antigen or nucleic acids in the cytoplasm of macrophages and dendritic cells in the depleted follicles in lymphoid tissues (*4*,*5*). Naturally occurring porcine circovirus–associated diseases is often accelerated or exacerbated by concurrent viral or bacterial infections, and secondary infections often occur as a result of immunosuppression (*6*).

Random nucleic acid amplification with or without prior enrichment for viral particle–associated nucleic acids (*7*,*8*), followed by deep sequencing and in silico similarity searches for sequences related to those of known viruses, have been highly productive in the field of animal virus discovery (*9*–*11*). We used this technique to identify virus sequences in affected tissues from companion animals with diseases of unknown cause. We identified a canine circovirus in the liver of a dog that had necrotizing vasculitis and granulomatous lymphadenitis, both of which are described in PCV2-infected pigs (*4*). We named this virus dog circovirus (DogCV) rather than canine circovirus to avoid confusion with the CaCV notation used for canary circovirus (*12*,*13*), canine calicivirus (*14*,*15*), and *Capsicum chlorosis* virus (*16*). A closely related variant of DogCV was sequenced independently in canine serum samples and was published recently (*17*); however, no disease association was described with the virus. To determine whether DogCV could be associated with canine vascular disease, we identified additional dogs with vascular and granulomatous lesions and examined the distribution of DogCV by ISH analysis. 

## Materials and Methods

### Animal Sample Collection

A 1-year-old castrated male dog that had been kenneled for 3 weeks was brought to the University of California, Davis (UC Davis), Veterinary Medical Teaching Hospital for evaluation of progressive vomiting and diarrhea with hematochezia. Despite initial supportive therapy at the referring veterinarian, clinical signs worsened; at UC Davis, the dog was treated for hypovolemic shock. Because of suspected disseminated intravascular coagulation and a poor overall prognosis, the owner elected to have the dog euthanized and granted permission for routine necropsy, which was performed at the Anatomic Pathology Service of the UC Davis School of Veterinary Medicine. The clinical and postmortem workups for infectious causes in this case included negative test results for infectious causes of enteric disease, such as canine parvovirus, canine enteric coronavirus, *Salmonella* spp., canine distemper virus, *Campylobacter* spp., *Clostridium perfringens* enterotoxin A gene, *Cryptosporidium* spp., and *Giardia* spp. Histologic results showed extensive fibrinoid vascular necrosis, thrombosis, and hemorrhage throughout the gastrointestinal tract and kidneys, as well as granulomatous lymphadentitis of the mesenteric lymph nodes. Special stains of histologic specimens revealed no detectable bacteria or other infectious agents. Liver tissue samples were collected, stored in whirl-pack bags, and frozen at −80°C until further processing.

### Sample Preparation and Nucleic Acid Extraction

A liver tissue sample (≈25 mg) were immersed in 1 mL cold Hank’s balanced saline solution and disrupted with a tissue homogenizer for 30 sec on ice. The resulting homogenates were placed on dry ice for 5 min and then thawed at room temperature (*18*). Freezing and thawing were repeated twice. Samples were clarified by centrifugation at 10,000 × *g* for 3 min; the supernatants were then filtered and underwent nuclease treatment as described (*19*). Viral nucleic acids were extracted by using the QIAamp Viral RNA Mini Kit (QIAGEN, Valencia, CA, USA) and stored at −80°C.

### Library Preparation and Sequencing

Viral nucleic acid libraries were prepared as described (*19*). The library of single-stranded DNA fragments was sequenced by using the Genome Sequencer FLX Instrument (Roche, Indianapolis, IN, USA).

### Sequence Data Analysis

The pyrosequencing reads were sorted and trimmed as described (*19*). Trimmed reads from each sample were assembled de novo by using the MIRA assembly program (*20*), with a criterion of >95% identity over 35 bp. The assembled sequences and singlets >100 bp were compared with the GenBank nonredundant nucleotide and protein databases (www.ncbi.nlm.nih.gov/genbank) by using BLASTn and BLASTx, respectively (http://blast.ncbi.nlm.nih.gov/Blast.cgi). Potential viral sequences with significant hits (E-value <0.001) to known virus sequences were identified.

### Genome Sequencing and Analyses

PCR and Sanger sequencing were used to confirm the presence of virus genome sequences assembled from deep sequencing reads. Inverse PCR was then used to amplify the genome of target circoviruses with primers based on the sequences obtained by deep sequencing. Virus genome sequences obtained were deposited in GenBank (accession nos. KC241982–KC241984). Putative open reading frames (ORFs) with coding capacity >100 aa were predicted by Vector NTI Advance 11 (Invitrogen, Carlsbad, CA, USA). The stem-loop structure was predicted by using Mfold (*21*).

### Phylogenetic Analysis

Phylogenetic analyses based on aligned amino acid sequences from full-length replicate (Rep) proteins were generated by using the neighbor-joining method in MEGA4 (*22*), using amino acid p-distances with 1,000 bootstrap replicates. Other tree-building methods, including maximum parsimony and maximum likelihood, were used to confirm the topology of the neighbor-joining tree.

### Prevalence Study of DogCV in Sample Cohorts

Real-time PCR using 2 primers and a conventional hydrolysis probe with a 5-primer 6-FAM and 3-primer TAMRA label and TaqMan Universal PCR Master Mix (Applied Biosystems, Foster City, CA, USA) was used to detect DogCV in DNA extracts from 3 dog sample cohorts: 1) fecal samples from 204 healthy dogs; 2) fecal samples from 168 dogs with diarrhea; and 3) blood samples from 480 dogs with thrombocytopenia and neutropenia, fever of unknown origin, or past tick bite. Primer pairs and probes for canine circovirus are given in the Table. Total nucleic acid was extracted by using the Corbett X-Tractor Gene platform (QIAGEN). Real-time PCR was conducted by using the real-time PCR instrument LightCycler 480 (Roche, Indianapolis, IN, USA) under these conditions: 50°C for 2 min, then 95°C for 10 min, followed by 40 cycles of 95°C for 15 sec and 60°C for 1 min. Synthetic DNA fragments (≈150 bp) of the corresponding regions were used to produce a standard curve and an analytical sensitivity of 10 molecules.

**Table:** Oligonucleotide primer pairs and probe for DogCV sequences*

Region and amplicon	Primers	Sequences, 5′→3′
Replicate gene, 66 bases	DogCV-forward	CTTGCGAGAGCTGCTCCTTATAT
	DogCV-reverse	CTCCACTTCCGTCTTCCAGTTC
	DogCV-probe	TCCGGAGATGACCACGCCCC
Capsid gene, 68 bases	DogCV2-forward	CTGTTGTGAAACTGAAAGAGACGAA
	DogCV2-reverse	TGACGTAGGTCTCCGGATACG
	DogCV2 -probe	AGCCTTGCCGCTGTCGCGTC

*DogCV, dog circovirus.

### ISH Analysis

A fourth sample cohort consisted of tissue samples from 21 necropsy cases of dogs whose clinical signs or microscopic lesions matched the sentinel animal (i.e., hemorrhagic diarrhea, vasculitis, and/or granulomatous disease); these samples were selected from the tissue archives of Anatomic Pathology at the UC Davis Veterinary Medical Teaching Hospital. Control tissues were obtained from 5 dogs whose cause of death was unrelated to vascular disease. Tissue sections chosen for analysis were, in part, case dependent because the presence of vasculitis or inflammation among these cases was not limited to a single tissue type. Spleen, lymph node, jejunum, and ileum were examined in all cases; other examined tissues were kidney, brain, adrenal gland, pancreas, duodenum, heart, and lung. Tissues from the sentinel dog were included in this analysis.

Tissue sections were mounted on 3-aminopropyltriethoxylane–coated slides (Fisher Scientific, Pittsburgh, PA, USA). A 25-nt oligomer (CTCAGACAGAGACACCGTTGCTATG) complementary to a segment of ORF2 (capsid) was 3′-end labeled by the addition of a single digoxigenin-II-dideoxy undine (Eurofins MWG Operon, Huntsville, AL, USA). A manual capillary-action work station (Fisher Scientific) was used to perform colorimetric DNA in situ hybridization. Tissue sections were deparaffinized and digested by incubation at 37°C for 10 min in 0.25% pepsin in 1× Tris-buffered saline (pH 2.0); pepsin activity was stopped by a 5-min incubation at 105°C. Nucleic acid was denatured by 5-min incubation at 105°C in 100% formamide. Tissue sections were then incubated at 37°C in 10 µmol of the digoxigenin-labeled probe in hybridization buffer (22.5% deionized formamide, 7.5% chondroitin sulfate, 5× saline sodium citrate, 0.25% blocking reagent, and 50 mmol phosphate buffer). Sections were incubated in an antidigoxigenin antibody solution (500:1 dilution) containing 2.5 mL buffer 1 with 5 μL of antidigoxigenin Fab fragments conjugated with alkaline phosphatase (750 U/mL) (Roche). Sections were then washed and developed according to manufacturer instructions before counterstain with 1% fast-green FCF for 5 min. Slides were mounted with ImmunoHistoMount (Immunobioscience, Mukilteo, WA, USA) and coverslipped with SHUR/Mount (Triangle Biomedical Sciences, Durham, NC, USA). No background hybridization was seen when replicate tissue sections were incubated with an unrelated digoxigenin-labeled probe with similar guanine-cytosine content or when matched tissue from unaffected dogs were incubated with the DogCV-specific probe (*23*–*25*).

### Electron Microscopy

Selected pieces of formalin-fixed lymph node tissue from the sentinel dog were post fixed in 2.0% glutaraldehyde and then routinely processed and embedded in epoxy resin (Eponate12 kit; Ted Pella, Inc., Redding, CA, USA). Selected thick sections were stained with toluidine blue, as described (*26*). Ultrathin sections from select areas of the lymph node were examined by using a Zeiss (Gottingen, Germany) 906E transmission electron microscope.

### Metagenomic Identification of Canine Circovirus

Of ≈10,000 sequence reads, 5 contigs and singlets composed of 52 sequence reads from the liver tissue had significant similarity to the Rep protein of circoviruses (E-value <1^−10^). Two bocavirus sequences were also detected. Because circoviruses have a circular genome, the full viral genome was then amplified by inverse nested PCR and the amplicon sequenced by primer walking. The assembled genome was named DogCV strain UCD1 (DogCV-UCD1).

## Results

### Genome Analysis and Phylogenetic Relationship

**Figure 1:**
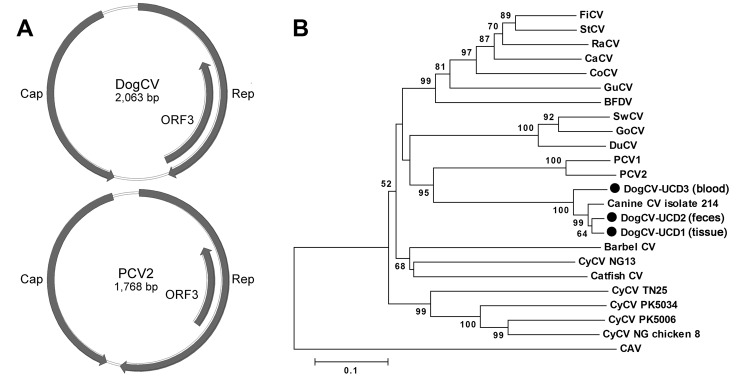
A) Genome organization of dog circovirus (DogCV) and porcine circovirus 2 (PCV2). B) Phylogenetic analysis of DogCV strains (UCD1–3, isolated from tissue, feces, and blood respectively) based on the amino acid sequence of the replicate (Rep) protein. GenBank accession numbers for circoviruses used in the analysis: Finch circovirus (FiCV), DQ845075; Starling circovirus (StCV), DQ172906; Raven circovirus (RaCV), DQ146997; canary circovirus (CaCV), AJ301633); Columbid circovirus (CoCV), AF252610; Gull circovirus (GuCV), DQ845074; beak and feather disease virus (BFDV), AF071878; Cygnus olor circovirus (SwCV), EU056310; Goose circovirus (GoCV), AJ304456; Duck circovirus (DuCV), DQ100076; Porcine circovirus 1 (PCV1), AY660574; PCV2, AY424401; canine CV, JQ821392; Barbel CV, GU799606; Cyclovirus (CyCV) NG13, GQ404856; Silurus glanis circovirus (Catfish CV), JQ011378; CyCV TN25, GQ404857; CyCV PK5034, GQ404845; CyCV PK5006, GQ404844; CyCV NG Chicken8, HQ738643); and chicken anemia virus (CAV), M55918.

The complete circular genome of DogCV-UCD1 was 2,063 nt (GenBank accession no. KC241982). Analysis of the genome sequence showed characteristics typical of circoviruses, including an ambisense organization with 2 major inversely arranged ORFs encoding the putative replication-associated (Rep, 303 aa), and capsid (Cap, 270 aa) proteins. A characteristic stem-loop structure with a conserved nonanucleotide motif (5′-TAGTATTAC-3′, similar to the consensus of bird and pig circoviruses) was also found in the 5′-intergenic region (135 nt, between the start codons of the 2 major ORFs). The 3′-intergenic region of DogCV-UCD1 between the stop codons of the 2 major ORFs was 203 nt (Figure 1, panel A) (*27*). 

The complete genome of another DogCV strain (DogCV-UCD2, GenBank accession no. KC241984) was amplified and sequenced from a fecal sample from a shelter-housed dog that had vomiting and diarrhea. This strain shared 95% overall genome nucleotide identity with DogCV-UCD1, and both strains showed 96%–97% nucleotide identity to the recently reported canine circovirus isolate 214 from blood (*17*).

The putative Rep proteins of DogCV-UCD1 showed 42%–54% amino acid identity to the Rep proteins of porcine and avian circoviruses, with the closest identity to PCV1. The DogCV-UCD1 capsid showed <30% amino acid identity to known circovirus capsids. Sequence alignment of the putative Rep protein of DogCV-UCD1 with those of known species in the genus *Circovirus* identified several highly conserved amino acid motifs, including WWDGY, DDFYGW, and DRYP. Motifs associated with rolling circle replication (FTLNN, TPHLQG, and CSK) and the dNTP binding (GXGKS) were also identified. The N terminal region of the Cap protein was highly basic and arginine-rich, as is typical for circoviruses. A phylogenetic analysis of the complete Rep protein of DogCV strains and all known circoviruses was performed, with chicken anemia virus (genus *Gyrovirus*) as the outgroup (Figure 1, panel B). The phylogenetic tree showed that DogCV strains grouped with PCV1 and PCV2, forming a distinct clade of mammalian circovirus, whereas circoviruses affecting birds clustered separately.

### Prevalence Study

DogCV was detected by real-time PCR in fecal samples from 14/204 healthy dogs and 19/168 dogs with diarrhea; the difference in prevalence was not significant (11.3% vs. 6.9%; p>0.05 by χ^2^ test). Of the 19 dogs with diarrhea who had DogCV detected in fecal samples, 13 (68%) were co-infected with >1 other pathogens, including canine enteric coronavirus, *Cryptosporidium* spp., *C. perfringens* α toxin, *Giardia* spp., *Salmonella* spp., *Campylobacter jejuni*, and *Campylobacter coli* (tested by PCR). 

The prevalence of DogCV in blood samples from the cohort of dogs with thrombocytopenia and neutropenia, fever of unknown origin, or past tick bite was 3.3% (16/480), similar to that reported for canine serum samples (2.9%, 6/205) (*17*). The partial Rep and/or Cap protein regions (≈350 bp) were amplified from 11/16 samples. All showed >96% nucleotide identity, except 1 amplicon, which had <90% nucleotide distance to DogCV-UCD1 and -UCD2. We sequenced the complete genome of this virus (DogCV-UCD3; GenBank accession no. KC241983); it showed 91%–92% amino acid identity of the complete Rep and Cap proteins to DogCV-UCD1 and -UCD2 and to the published canine circovirus (CaCV-1 strain NY214; GenBank accession no. JQ821392) (*17*). 

### ISH Analysis and Pathologic Findings in Positive Cases

To establish tissue distribution and investigate whether DogCV contributes to canine disease, we developed and validated an ISH oligomeric probe and examined the sentinel dog and dogs from 21 suspected, retrospective cases that included >2 of these 3 signs: vasculitis, hemorrhage, or granulomatous disease. A wide spectrum of affected tissues was represented in this group; matching tissues were also examined from 5 control dogs in which these signs were not present. Samples from the sentinel dog (dog 1) and 3 other dogs (dogs 2–4) were positive for DogCV by ISH analysis. All other tissue samples from control dogs were negative by ISH analysis. Clinical signs, gross and histologic findings, and distribution of virus DNA as determined by ISH were used to examine a possible causal role for DogCV. 

Dog 1 was a male beagle who had acute onset of vomiting and hemorrhagic diarrhea. Dog 2 was a 5-year-old, female, spayed Boston terrier who had vomiting and diarrhea. Dog 3 was a 1-year-old, female, spayed boxer with a 5-day history of lameness and progressive tetraparesis. Dog 4 was a 2-year-old Greyhound found dead with bicavitary hemorrhage; blood smear and PCR showed this dog was co-infected with *Babesia conradae*. 

**Figure 2:**
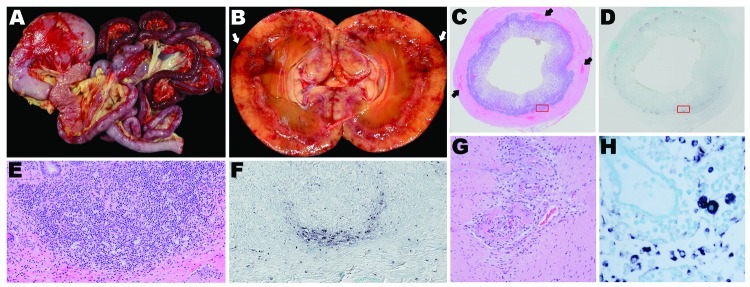
Organ tissues from sentinel dog (dog 1) and 2 other dogs (dogs 2 and 3) that were positive by in situ hybridization (ISH) analysis for dog circovirus (DogCV). A) Gross view of the gastrointestinal system from dog 1. Multifocal to coalescing hemorrhages are shown in the stomach and intestinal serosa. B) Gross view of the kidney from dog 2. Segmental regions of hemorrhage and necrosis (infarction) can be seen within the cortex and radiating from the medullary papilla to the capsule. C) Hemolyxin and eosin (H&E) stain of the ileum from dog 1. Peyer’s patches are moderately depleted, and multifocal regions of hemorrhage are present within the muscular intestinal wall. D) ISH of the ileum from dog 1. Peyer’s patches circumferentially contain abundant DogCV DNA. E) H&E stain of a Peyer’s patch in the ileum from dog 1, showing moderate depletion of lymphocytes and an increased population of macrophages within the germinal center and the peripheral base of the follicle. F) ISH of a Peyer’s patch in the ileum from dog 1. DogCV DNA is rich within the cytoplasm of abundant cells at the periphery of the follicle, and individual cells are scattered within the germinal center, lymphatic channels, and submucosa. The positive cells have the morphologic appearance of macrophages. G) H&E stain of the jejunum from dog 3, showing segmental, circumferential, fibrinoid necrosis of the artery. H) ISH of a mesenteric lymph node from dog 2. Macrophages in the medullary sinus and lymphatic cords contain abundant DogCV DNA.

Gross examination revealed consistent lesions among these 4 dogs, including lymphadenopathy and hemorrhage. In dogs 1 and 2, the hemorrhage was associated with the gastrointestinal tract (Figure 2, panels A, C); dog 2 had additional multifocal to coalescing regions of hemorrhage in the kidneys (Figure 2, panel B). Gross evidence of hemorrhage in dog 3 was restricted to the ventral surface of the brain along the basilar artery overlying the medulla; dog 4 had bicavitary hemorrhage. The common histologic lesion in all dogs was fibrinonecrotizing vasculitis, although the distribution of affected vessels and the amount of associated hemorrhage varied. In dog 1, segments of inflamed or necrotic vessels were seen in the intestine (multiple segments), urinary bladder, liver, spleen, and lungs. In dog 2, vasculitis was limited to the intestine and spleen, and in dog 3, vasculitis was in kidneys, intestine (Figure 2, panel G), heart, liver, spleen, and meninges. In dog 4, only a few vessels were affected, at the corticomedullary junction in the kidneys. For all dogs, histiocytic drainage or granulomatous lymphadenitis were seen in Peyer’s patches (Figure 2, panels C, E) and in >1 lymph node. In addition, in dogs 2 and 3, multiple lymph nodes were severely necrotic. All dogs had microscopic lesions in the kidneys, but intensity and character varied widely. Dogs 1 and 2 had tubular necrosis with little inflammation, dog 3 had a severe granulomatous interstitial nephritis, and dog 4 had multifocal hemorrhage and minimal lymphocytic, plasmacytic nephritis. Dogs 1 and 3 had multifocal pancreatitis and adrenalitis. No multinucleate giant cells, common in pigs infected with PCV2, were seen.

By ISH analysis, abundant cytoplasmic viral nucleic acid was detected in macrophages within germinal centers and subcapsular and medullary sinuses of the mesenteric lymph nodes from all 4 dogs (Figure 2, panel H), mandibular lymph nodes from 2 dogs, ileal Peyer’s patches from all 4 dogs (Figures 2, panels D, F), ellipsoids (terminal arterioles) of the spleen for 1 dog, and germinal centers in splenic white pulp for 2 dogs. Although the reticular network was abundantly positive in dog 1 (Figure 2, panel H), the common pattern was localization to the centers of lymphoid or Peyer’s patch follicles (Figure 2, panels D, F), corresponding to the morphology and distribution of dendritic cells. Rare nuclei of elongate cells (presumed endothelium) lining small vessels of the adrenal cortex or intestinal lamina propria suprajacent to the Peyer’s patches were positive in 2 dogs. No nucleic acid was detected by ISH analysis in other tissues, regardless of the character or intensity of the inflammation. None of the 5 control dogs showed DogCV DNA by ISH.

### Transmission Electron Microscopy Analysis

**Figure 3:**
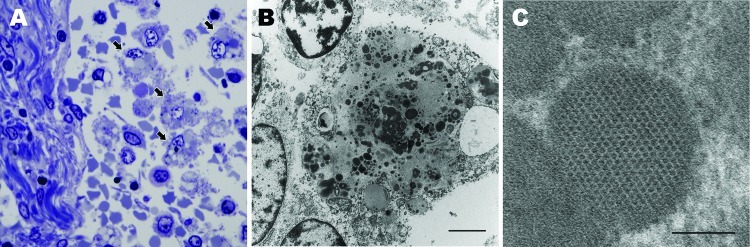
Lymph node from sentinel dog from which dog circovirus was identified. A) Toluidine blue stain shows multiple macrophages within the medullary sinus contain vacuoles and discrete, oblong to round, variably stained cytoplasmic bodies (arrows). B) A single macrophage adjacent to a lymphocyte (upper left) and partial profiles of other cells. Intracytoplasmic inclusion bodies are distributed throughout the macrophage cytoplasm, along with mitochondria and vacuoles. Scale bar indicates 2 µm. C) Intracytoplasmic inclusion bodies contain granular content and sometimes paracrystalline to herringbone arrays of 10–11 nm diameter viral-like particles. Scale bar indicates 100 nm.

Ultrastructural analysis of the mesenteric lymph node from dog 1 revealed macrophages laden with large numbers of intracytoplasmic inclusions (Figure 3, panel A). Inclusions were round, oblong, or irregular; were <0.5 µm; and often clustered (up to 25 per cell) within the cytoplasm, (Figure 3, panel B). Most inclusions were granular and electron-dense and had a distinct periphery but were nondelineated by membrane. Some inclusions contained paracrystalline arrays of icosahedral virions that were 9–11 µm in diameter (Figure 3, panel C).

## Discussion

We identified a novel circovirus, DogCV, in the liver of a dog that had necrotizing vasculitis and granulomatous lymphadenitis. We characterized the genome of multiple DogCV strains, determined DogCV prevalence in dog fecal and plasma samples and tissue distribution in infected animals, and detected paracrystalline arrays in inclusion bodies in macrophages. Real-time PCR analysis showed a prevalence of 11.3% and 6.9% in fecal samples from dogs with diarrhea and healthy dogs, respectively. DogCV DNA was also found in 3.3% of blood samples from dogs with thrombocytopenia and neutropenia, fever of unknown origin, and past tick bite, which is approximately the same percentage as previously reported (*17*).

ISH analysis of the sentinel dog and 21 additional dogs selected retrospectively from past necropsies detected viral nucleic acid in 4 dogs, including the sentinel dog, and the histopathologic features and distribution of virus in tissue samples from these dogs were evaluated. The organs affected varied even in this small set of animals, but all dogs had necrotizing vasculitis and hemorrhage, and all but 1 had lymphadenitis and granulomatous disease. Because the tested retrospective animals were chosen to match the sentinel case, our sampling is biased, and the spectrum of diseases associated with this virus might be broader than we detected. Among dogs positive by ISH, disease signs varied, and clinical, gross, and microscopic features in some of the disease syndromes were similar to those associated with PCV2 infection (*4*,*5*). In particular, porcine dermatitis and nephropathy syndrome shares many of the histologic features of seen in DogCV-positive dogs (*28*,*29*), and PCV2 has been reported to cause necrotizing lymphadenitis, vasculitis, or neurologic disease (*4*,*30*,*31*). Virus distribution, as assessed by ISH analysis, is also similar between DogCV and PCV2. 

Viral DNA was consistently detected in the cytoplasm of macrophages and monocytes in lymphoid tissues of infected dogs. Virus distribution in pigs infected with PCV2 is most consistently within lymphoid tissue, with sporadic reports of virus in other tissues (*4*,*23*,*32*,*33*). For example, in pigs with dermatitis and nephropathy syndrome, granulomatous inflammation of the kidneys is commonly reported; however, virus is detected in renal tissue in only a few cases by any method. In our study, DogCV DNA was detected in lymphoid tissues, including Peyer’s patches, even for dogs 3 and 4, where no clinical or histologic enteric lesions were detectable. DogCV DNA was also found in small, end-capillary endothelial channels of the intestinal lamina propria and the adrenal cortex; however, confirmation of these cells as endothelial will require further investigation. 

Two distinct histologic features of PCV2 infection are viral inclusions and multinucleate giant cell formation (*34*), neither of which was detected by routine histology in the dogs infected with DogCV. By electron microscopy, however, macrophages within the lymph node contained abundant cytoplasmic viral inclusions composed of dense granular or paracrystalline arranged virus. Affected cells were found in both the sinus and medullary cords of the mesenteric lymph node examined. Ultrastructural inclusions were similar to a subset of cytoplasmic inclusions that have been described in PCV2 infected tissues (*35*,*36*).

Numerous experimental and natural disease studies have indicated that PCV2 infection most often leads to clinical diseases in the presence of co-infection with other swine pathogens. PCV2 enhances viral (e.g., porcine parvovirus, porcine reproductive and respiratory syndrome virus), bacterial, protozoal, metazoal, and fungal infections in pigs (*6*). Among the dogs with diarrhea in our study, most (68%) of those positive for DogCV had co-infection with >1 enteric pathogens. Also, for the small set of cases in which we identified virus in situ, 1 dog was co-infected with a canine bocavirus and 1 with *Babesia conradae*. The role of co-infection in the pathogenesis of disease in these cases is unclear.

In summary, DogCV should be considered in cases of unexplained vasculitis in dogs, although further studies will be required to ascertain whether and when DogCV causes disease. DogCV also could be a complicating factor in other canine infectious diseases, as is the case with PCV2, which is most dangerous in pigs co-infected with other pathogens. Future research into the contribution of DogCV to disease should carefully consider these potential viral and host factors.
